# Human immune responses against *Shigella* and enterotoxigenic *E. coli*: Current advances and the path forward

**DOI:** 10.1016/j.vaccine.2017.05.034

**Published:** 2017-05-27

**Authors:** Monica A. McArthur, Milton Maciel, Marcela F. Pasetti

**Affiliations:** a Department of Pediatrics, Center for Vaccine Development, University of Maryland School of Medicine, Baltimore, MD, USA; b Enteric Diseases Department, Naval Medical Research Center/ETEC Vaccine Program, Silver Spring, MD, USA; c Henry M. Jackson Foundation for the Advancement of Military Medicine, Bethesda, MD, USA

**Keywords:** *Shigella*, ETEC, Human immunology, Vaccines

## Abstract

Robust and well-established immunological assays and firm immune correlates of protection that can predict disease outcome and/or vaccine efficacy are essential to adequately assess human immune responses to infection and vaccination. The availability of reagents and calibrated controls is also critically important to standardize assays and generate comparable results among different laboratories. The workshop “Human Immune Responses against *Shigella* and ETEC: Current Advances and the Path Forward” held during the VASE meeting provided an opportunity to disseminate and discuss recent advances in the field *of Shigella* and ETEC immunology, identify research needs, and propose collaborative activities to advance the field. Four presentations featured current knowledge on humoral and cellular immune responses to *Shigella* and ETEC during infection and vaccination. A discussion followed on immunological methods relevant for clinical studies, immune parameters associated with protection, harmonization of assays among laboratories, and availability of reagents and standards. Specific recommendations proposed to facilitate “the path forward” included supporting communication among scientists, harmonization of assays and sharing of protocols, the creation of a repository of reagents and calibrated controls and distribution of such material to the research community, and expansion of exploratory studies to better understand the interactions between these pathogens and the human immune system and the ensuing responses.

## 1. Introduction

*Shigella* and ETEC are among the top attributable causes of severe diarrheal and dysenteric illness in young children in the developing world, and in individuals traveling to endemic areas, including military personnel. There are currently no licensed vaccines available. Our limited knowledge of the immunological mechanisms that protect the host against these pathogens and relevant immunological parameters that can predict disease outcome and/or vaccine efficacy has hindered progress in vaccine development. Elevated levels of serum antibodies recognizing the *Shigella* lipopolysaccharide (LPS) O-antigen have been associated with reduced incidence of shigellosis caused by serotype-matching strains in naturally exposed individuals [1,2] and recipients of *Shigella* O-polysaccharide conjugate vaccine candidates [3,4]. LPS-specific IgA Antibody Secreting Cells (ASC) [5] and IpaB-specific IgA B memory cells [6] have also been proposed as putative indicators of protective immunity. Antibodies against *E. coli* colonization factors (CF), coli-surface antigens (CS), and the heat-labile toxin (LT), have been linked to reduced risk of ETEC diarrhea [7,8]. A series of clinical trials (ongoing and forthcoming) are set to address elusive immune correlates of protection against *Shigella* and ETEC in the context of natural infection and controlled experimental infection (ClinicalTrials.gov: NCT01638039, NCT02646371, NCT03038243, NCT02773446, NCT01922856). The information derived will have a substantial impact in the field and in the current vaccine development landscape.

Accurate evaluation of human immune responses to infection and vaccination requires well-established immunological assays. The harmonization of methods and the establishment of calibrated controls are equally important to generate robust data that can be compared across laboratories and among studies. This workshop provided a forum to disseminate and discuss recent advances in the field of *Shigella* and ETEC immunology, identify research needs, and propose collaborative activities that can advance the field. Presentations on humoral and cellular immune responses to *Shigella* and ETEC infection and vaccination were followed by discussions on relevant methods to evaluate immune responses in clinical studies, measurements that could predict disease outcome and/or protection after infection and vaccination, harmonization of assays among laboratories, and a repository of reagents and controls. Specific recommendations were proposed to strengthen research efforts.

## 2. Functional and antigen-specific serum antibodies induced upon *Shigella* infection and vaccination

In the first presentation, Dr. Marcela Pasetti, from the Center for Vaccine Development, University of Maryland School of Medicine, described the development of quantitative assays to measure *Shigella*-specific serum bactericidal (SBA) and opsonophagocytic killing antibody (OPKA) activity. Her group examined SBA and OPKA responses, along with Ipa-, VirG-, and LPS2a-specific serum IgG responses in volunteers immunized with a *Shigella* vaccine candidate, EcSf2a-2, and in unvaccinated controls before and after these individuals were challenged with virulent *S. flexneri* 2a [9]. Prechallenge antibody titers (SBA and OPKA, as well as IpaB- and VirG-specific IgG) were found to be significantly associated with clinical protection and reduced severity of disease post-challenge. The SBA assay detected more responses post-challenge and post-vaccination. SBA and OPKA responses were also detected in human adult volunteers after a single oral immunization with live attenuated *Shigella* vaccine strains CVD 1204 and 1208S [9]. The specificity of these functional bactericidal and opsonophagocytic antibodies remains to be determined. Nahm et al. reported SBA activity of mouse monoclonal anti-*Shigella* O-polysaccharide (OPS) antibodies [10]. Riddle and colleagues reported SBA responses in human volunteer recipients of Flexyn2a, a vaccine consisting of *S. flexneri* 2a O-antigen biologically conjugated to the exotoxin protein A of *Pseudomonas aeruginosa* (EPA) [11]. Dr. Pasetti reported that in studies performed by her group, LPS antibody competition and depletion significantly reduced but did not totally abrogate SBA activity, suggesting involvement of other antigens. These encouraging results warrant further studies to establish the capacity of these assays to predict protective immunity and vaccine efficacy. The identification of serological correlates of protection is indeed critically important to inform vaccine design and to facilitate clinical development efforts.

## 3. Immune profile of vaccine-induced immunity against *Shigella dysenteriae* type 1

The second presentation, by Dr. Marie-Lise Gougeon, Institut Pasteur (Paris, France), described studies performed by her team aimed to identify a molecular immune signature of responders to the live attenuated *S. dysenteriae* type-*1* vaccine candidate SC-599, which harbors deletion of intracellular spreading [icsA], iron chelation [ent, fep], and shiga toxin A subunit [stxA] genes. The vaccine was ingested by healthy adult volunteers in doses of 10^5^ CFU and 10^7^ CFU, who along with placebo controls were examined for LPS-specific serum antibody and ASC responses. Both 10^5^ and 10^7^ CFU doses induced significant IgA and IgG LPS-specific ASCs and serum antibody responses, comparable in magnitude to those induced by other vaccine strains that prevented illness following experimental challenge [12]. An extensive cytokine profile analysis was conducted in culture supernatant from vaccine or placebo recipients’ peripheral blood mononuclear cells (PBMC) stimulated overnight with *S. dysenteriae* type-1 LPS. A total of 25 cytokines and chemokines were measured by Luminex, the majority of which were detected except for IL-4, IL-5, IL-7, and IL-13. Vaccine responders in the 10^5^ CFU dose group (but not placebos) had elevated levels of IL-17 nine days after immunization, as compared to baseline. Responders in this group also exhibited elevated levels of IL-1β, IL-6, TNF-α, G-CSF, and IFN-γ. Some cytokines, such as IL-2 and IL-9, were not induced in response to this vaccine. The cytokine profile identified in the 10^5^ CFU vaccine responses corresponded to a Th1/Th17 signature. This is the first demonstration of Th17 cytokine responses to *Shigella* in humans, which is in agreement with the *Shigella*-specific Th17 profile induced in mice following infection [13]. Furthermore, positive correlations were found between the ASC frequency and levels of IL-6, G-CSF, and IFN-γ. The profile of responders to vaccine SC-599 included: (1) LPS-specific IgG and IgA ASC, and Th1 and Th17 responses; (2) association of IgA-producing cells with production of IL-6, G-CSF, and IFN-γ; 3) IL-6, IL-10, G-CSF, and IFN-γ secretion. These results support the notion of a distinct immunological T cell signature associated with responders that is worthy of exploring in upcoming clinical vaccine studies, particularly those using other *Shigella* antigens and immunization routes.

## 4. Humoral immune responses to ETEC infection and vaccination

The third presentation, given by Dr. Subhra Chakraborty from the Department of International Health of the Johns Hopkins Bloomberg School of Public Health, Baltimore, MD, described mucosal immune responses to ETEC in volunteers orally challenged and re-challenged with strain H10407, which expresses the CFA/I colonization factor, the heat-labile (LT) and heat-stable (ST) toxins, and O78:H11 LPS [14]. She presented the association of severity of diarrhea and immune responses in a dose de-escalation study. Anti-LPS antibody responses were most frequent, followed by anti-LTB and anti-CFA/I in serum, lymphocyte supernatants (ALS), fecal extracts, and saliva; the rates of seroconversion were higher for IgA than for IgG, reflecting the mucosal challenge. The re-challenged volunteers were 90% protected from diarrhea and showed blunting of the serological responses to the antigens evaluated. Blunting was also observed for IgA in ALS [15] and stool [14]. Dr. Chakraborty also described serum and mucosal (fecal and ALS) immune responses in volunteers orally immunized twice (on days 0 and 21) with the attenuated ETEC vaccine ACE527, which contains CFA/I, CS1, CS2, CS3, CS5, and CS6 colonization factors along with the B subunit of LT (LTB) [16,17]. ALS IgA responses were observed one week after ACE527 vaccination, although at lower levels following the second dose. The serological responses in volunteers challenged with H10407 or immunized with ACE527 had similar profiles except for a higher frequency of anti-LTB responses among vaccine recipients, likely due to the greater LTB content of the vaccine. Dr. Chakraborty presented an innovative approach to improve mucosal antibody measurements, by which LPS-, CFA/I-, and LTB-specific IgA were determined in the first “induced” stool (FIS) of H10407 challenged volunteers [14]. This first stool was induced through slow ingestion of a bowel cleansing solution (polyethylene glycol 60 g/L, GoLytely^®^) that continued only until the first liquid sample (FLS) was obtained. There was good agreement among ETEC-specific IgA levels measured in FIS, FLS, and fecal extracts from spontaneous samples, warranting further investigation of these methods in future studies. In the last part of her presentation, Dr. Chakraborty presented a comparison of immunological assays and frequency of responses, and reported that ALS was the most sensitive in detecting the highest number of responders to the different antigens following infection and re-infection [14], an observation also made by others [18]. Importantly, there was a strong correlation between ALS and serum IgA titers in naïve infected volunteers, but not in those re-challenged.

## 5. Cellular responses following ETEC challenge

The fourth presentation of the workshop was given by Dr. Monica A. McArthur from the Center for Vaccine Development, University of Maryland School of Medicine. This presentation focused on the cell mediated immune (CMI) responses in volunteers challenged with wild-type ETEC strain H10407. Antibodies have been proposed as a major contributor to protection due to the non-invasive nature of ETEC; however, few studies have investigated the potential contribution of T cells and their role in protection against this organism. Dr. McArthur and her team explored ETEC-specific total CD4+ T cell responses following wild-type ETEC challenge [19]. She reported no significant difference in total CD4+ T cells between volunteers who developed moderate to severe diarrhea (susceptible) and those who did not (resistant). However, significantly higher ETEC-specific production of TNF-α and IL-2 by multifunctional CD4+ T cells from resistant volunteers were detected. Furthermore, higher levels of integrin α4β7 expression by CD4+ T cells from resistant volunteers were identified. Subsets of CD4+ T cells expressing, or not, the gut-homing molecule integrin α4β7 contribute to ETEC-specific cytokine production. These preliminary results indicate a potential role for ETEC-specific cytokine production by CD4+ T cells that home to the gut as well as extra-intestinal sites in resistance to ETEC infection. Dr. McArthur also presented data regarding the gut-homing potential of peripheral T follicular helper cells (pT_fh_) following ETEC challenge [19]. pT_fh_ are a circulating subset of CD4+ T cells which are responsible for providing B cell “help”, and the homing potential of this subset has been associated with differential antibody production by B cells [20]. Higher levels of expression of the gut-homing molecule integrin α4β7 by pT_fh_ were demonstrated in volunteers who did not develop moderate to severe diarrhea. Furthermore, integrin α4β7 expression by pT_fh_ was inversely correlated with stool output in volunteers post-challenge. Additionally, there was a correlation between higher expression of integrin α4β7 by pT_fh_ and higher ETEC-specific IgA B_M_ responses. Taken together, these results indicate that the gut-homing potential of pT_fh_ may be an important indicator of protection against ETEC and that the presence of pT_fh_ with gut-homing potential prior to challenge may play a role in development of IgA B_M_ responses at later time-points, suggesting that it might be an early indicator of long-term protection.

## 6. Summary of the group discussions

The *Shigella* functional antibody assays generated much interest, with multiple questions and comments regarding antigens recognized by these antibodies (LPS is a bactericidal target but other antigens might be involved as well), type of immunoglobulin involved (IgG vs. IgM), cells involved (B and supporting T cells?), whether these antibodies actually play a role in protection and their location of action in vivo, the ability of *Shigella* and ETEC to bind complement proteins (factor H and C4b binding protein) and the source of complement in SBA assays (e.g. human, guinea pig, rabbit), the stability of target strains (more problematic for *S. sonnei*), the sensitivity and miniaturization of assays for use in the pediatric population, and whether antibodies with these antimicrobial functions could be detected in the gut. Dr. Rubhana Raqib mentioned that her group successfully detected bactericidal antibodies in context of natural infection [21,22] and more recently in response to *S. sonnei* (WRSS1) vaccination (ClinicalTrials.gov: NCT02934178).

Dr. Pasetti explained that her group and Dr. Kaminski's group at Walter Reed Army Institute of Research (WRAIR) are working with Dr. Moon Nahm, an expert in functional antibody assays from University of Alabama (UAB), to standardize the *Shigella* functional assays developed in their laboratories. Dr. Nahm will independently assess the reproducibility of these assays and will assist in optimizing and harmonizing procedures, and establishing calibrated controls that will facilitate comparison of results from different laboratories. Other vaccine research groups are pursuing multiplex technology to measure *Shigella* functional antibody activity [23]. Ultimately, the idea is to establish robust and practical assays that can be widely used by the community. The value of functional antibodies as predictors of protective immunity will be better understood as they continue to be studied in the context of natural and vaccine-induced immunity.

A good part of the workshop discussion centered on the selection, value, and practicality of different methods to evaluate *Shigella* and ETEC immune responses post-vaccination and post-challenge in clinical studies, specimens typically obtained for immunological measurements, the importance of timing (e.g. early analysis of cytokines) and sample quality to ensure accurate results. There was agreement that the immune responses to be examined will inevitably vary based on the vaccine concept, route of administration, vaccination schedule, and population involved (primed vs. un-primed). The panel also discussed differences between clinical studies in unexposed populations and those in endemic areas, along with the need for assays that would allow the detection of immune responses in the presence of preexisting immunity. For humoral responses, Dr. Pasetti mentioned that the field is moving away from traditional ELISAs into multiplex technology, which allows the simultaneous analysis of antibody responses to multiple antigens, and functional assays that provide information on actual anti-microbial activity, as opposed to simple antibody binding assays that measure all types of antibodies. Dr. Gougeon's presentation highlighted the advantage of analyzing immune responses in aggregate (e.g., antibodies, ASC, and cytokines) to identify signatures or distinct immune profiles that might be indicative of vaccine performance.

State-of-the-art knowledge on *Shigella* and ETEC human immune responses and the application of traditional assays were highlighted in the selected presentations. Dr. McArthur proposed that in addition to the standard assays, exploratory studies (particularly T cell assays) continue to be critical to identify predictors of disease outcome and protection after infection and vaccination and to ultimately selecting the appropriate assay repertoire. For cell-mediated immunity, the use of whole blood was proposed as a convenient alternative to PBMC, which can be more challenging to obtain. A number of suggestions were offered for accurate determination of mucosal IgA, including the evaluation of both antigen-specific and total IgA, and the calculation of a ratio specific/total IgA to reduce variation—although with the cautionary note that total mucosal IgA may be elevated due to vaccination and infection, in which case the ratio would reduce the power to detect antigen-specific responses. The normalization of IgA titers to total proteins in mucosal samples was suggested to avoid this potential pitfall. It was stated that IgA levels in fecal samples are affected by specimen preservation, consistency, and efficiency of antibody extraction, among other technical challenges. Whole gut lavage (WGL) was mentioned by Dr. Chakraborty as an alternative to fecal samples for mucosal antibody measurements [24]; WGL can be obtained in a controlled manner and might better represent the small bowel content where ETEC infection takes place. An obvious disadvantage of this procedure is its invasiveness, particularly in pediatric studies. A final suggestion from the panel was to seek support to facilitate communication and sharing of information and materials among scientists in the field, possibly using blogs and social media for a rapid and convenient interaction.

## 7. The way forward

The panel emphasized the need for more interaction among members of the scientific community with the same research interests and centralization of efforts to facilitate exchange of information and materials. Specific recommendations proposed:

1. Harmonization of immunological assays among laboratories.
2. Sharing of Standard Operating Procedures (SOPs).
3. Expanding exploratory assays to identify immune correlates of protection.
4. Establishing a repository of reagents (antigens and calibrated controls).
5. Creating opportunities that will facilitate communication and sharing of information, such as a website, blogs, interest groups using social media, advocacy groups, and future workshops.

Several research groups, including investigators from WRAIR; UMB;, Johns Hopkins Bloomberg School of Public Health, UAB, and the International Center for Diarrheal Disease Research, Bangladesh (icddr,b) are working together on assay qualification and harmonization of SOPs, and exchange of reagents, calibrated controls, and test panel samples. Immunological assay development and refinements are being supported by PATH, the National Institutes of Health's (NIH) National Institute of Allergy and Infectious Diseases (NIAID), as well as other organizations, through various funding mechanisms. Dedicated financial and programmatic support would enable making assay procedures and calibrated reagents available to the community. Arguably, such investment would have a high return furthering our understanding of the interactions between these organisms with the human immune system and the ensuing responses, and accelerating the development of prophylactic and therapeutic tools. Future meetings and dedicated workshops will provide an opportunity to share knowledge and strategize efforts to achieve these goals.

### Disclaimers

The views expressed in this article are those of the authors and do not necessarily reflect the official policy or position of the Department of the Navy, Department of Defense, or the U.S. Government.
